# Biological tests carried out on serum/plasma samples from donors of human body material for transplantation: Belgian experience and practical recommendations

**DOI:** 10.1007/s10561-018-9721-2

**Published:** 2018-08-29

**Authors:** Elizaveta Padalko, Katrien Lagrou, Marie-Luce Delforge, Hilde Jansens, Nadine Ectors, Jean-Paul Pirnay, Johan Klykens, Etienne Sokal, Ludo Muylle, Agnes Libois, Alain Vanderkelen, Gilbert Verbeken, Conny Matthys, Dominique Goossens, Geert Hanssens, Muriel Baltes, Hilde Beele

**Affiliations:** 1 Department of Clinical Chemistry, Microbiology and Immunology, Ghent University/University Hospital, De Pintelaan 185, 2P8, 9000 Ghent, Belgium; 2 School of Life Sciences, Hasselt University, Agoralaan Building D, 3590 Diepenbeek, Belgium; 3 Working Group on Cells, Tissues and Organs of the Superior Health Council of Belgium, Brussels, Belgium; 4 KU Leuven and University Hospitals of Leuven, Herestraat 49, 3000 Louvain, Belgium; 5 Université Libre de Bruxelles/Hopital Erasme, Route de Lennik 808, 1070 Brussels, Belgium; 6 Antwerp University and Antwerp University Hospital, Wilrijkstraat 10, 2650 Edegem, Belgium; 7 Laboratory for Molecular and Cellular Technology, Queen Astrid Military Hospital, Bruynstraat 1, 1120 Brussels, Belgium; 8 Centre de Thérapie Cellulaire, Cliniques Universitaires St Luc, Université Catholique de Louvain, 10 av Hippocrate, B 1200 Brussels, Belgium; 9 CHU Saint-Pierre, Université Libre de Bruxelles, 322 rue haute, 1000 Brussels, Belgium; 10 Red Cross, Namur, Belgium; 11 Sint-Genesius-Rode, Belgium

**Keywords:** Reporting, Interpretation, Biological test, Donor of human body material

## Abstract

This paper on the biological tests carried out on serum/plasma samples from donors of human body material (HBM) is the result of a project of the working Group of Superior Health Council of Belgium formed with experts in the field of HBM and infectious serology. Indeed, uncertainty about the interpretation of biological test results currently leads to the sometimes unjustified cancelling of planned donations or the rejection of harvested HBM, whilst more sophisticated diagnostic algorithms would still allow the use of organs or HBM that would otherwise have been rejected. NAT tests will not be discussed in this publication. In the first part some general aspects as the need for a formal agreement between the Tissue Establishment l and the laboratory responsible for the biological testing, but also some specifications regarding testing material, the choice of additional biological tests, and some general aspects concerning interpretation and reporting are discussed. In a second part, detailed information and recommendations concerning the interpretation are presented for each of the mandatory tests (human immunodeficiency virus, hepatitis B virus, hepatitis C virus and syphilis) is presented. A number of not mandatory, but regularly used optional serological tests (e.g. for the detection of antibodies to *Toxoplasma gondii*, Epstein–Barr virus, human T cell leukemia virus and cytomegalovirus) are also extensively discussed. Although the project was meant to provide clarification and recommendations concerning the Belgian legislation, the majority of recommendations are also applicable to testing of donors of tissues and cells in other (European) countries.

## Introduction

The directive 2004/23/EC, 2006/17/EC, 2006/86/EC and annex 28 of EU mapping of blood donor testing requirements (2015) have been transposed in Belgian regulation.

The Royal Decree (RD) of 28 September 2009 setting standards of quality and safety for the donation, harvesting, procurement, testing, processing, storage and distribution of human body material that the banks for human body material, the intermediary structures and the production establishments must comply with, describes, among other things, the biological tests that need to be carried out among living and deceased donors.

For some serological parameters, interpreting their results may turn out to be a complex issue, giving rise to doubts and/or uncertainties.

Uncertainty about the interpretation of serological test results currently leads to the sometimes unjustified cancelling of planned donations or the rejection and destruction of harvested HBM, whilst more sophisticated diagnostic algorithms would still allow the use of tissues that would otherwise have been rejected.

In the current paper, members of the working group in “cells, tissues and organs of human and animal origin” of the Superior Health Council (SHC) have examined both the mandatory serological tests as defined in Royal Decree of 28th of September 2009, (antibodies against human immunodeficiency virus (anti-HIV)-1,2; Hepatitis B surface antigen (HBsAg), antibodies against Hepatitis B core antigen (anti-HBc), antibodies against Hepatitis C virus (anti-HCV) and the syphilis screening test) as well as optional serological tests (antibodies against cytomegalovirus (anti-CMV), antibodies against *Toxoplasma gondii* (anti-Toxoplasma), antibodies against Epstein–Barr virus (anti-EBV), antibodies against human T-cell leukemia virus (anti-HTLV)) in the process of selection of the HBM donors. Other potential optional tests, mainly those for the testing of specific infectious agents, non-endemic in Europe, in a temporary epidemiological context (e.g. West Nile virus, Chikungunya, Ebola, Zika are not discussed in the current paper). In all cases, this concerns optional tests, which are often only relevant during a specific period of time, i.e. when there is an increased prevalence of the infection in question and will imply the use of highly specialized tests performed in specific laboratories.

This paper does not deal with any other tests than those mentioned above. It focusses on the interpretation and reporting of serological tests carried out within the legal framework of the Royal Decree of 28 September 2009. It does not consider these tests for diagnostic purposes.

## General considerations

### Agreement between the institution involved in the donation, harvesting, procurement, testing, processing, storage and distribution of HBM and the laboratory responsible for the biological testing on samples from HBM donors (laboratory)

Prior to transferring the samples to the laboratory that will undertake the biological testing as part of the HBM donation process, an agreement needs to be signed between the HBM bank concerned and the laboratory. At the very least, the latter should clarify the methodology of the tests used by the laboratory, the expected turnaround time for the assays and the agreements related to the reporting of the results.

### Source material

The biological tests are carried out on donor serum or plasma. They are not to be performed on other fluids or secretions such as aqueous or vitreous humor, unless specifically justified clinically using a validated test for such other fluid (2006/17/EC). The biological tests cannot be performed on the HBM itself.

While venous puncture represents a standard blood collection method, use of arterial and intracardiac blood can be justified (Baleriola et al. 2012; Kitchen et al. 2013).

Blood samples may come from living or deceased donors In case of a living donor, the sample should be collected at the time of or shortly after donation (max. 7 days). This implies the possibility to collect and test an extra sample or retest on a later time point if needed. In case of a deceased donor, this possibility does not exist

The blood samples must have been taken within 48 h prior to death. If this is not possible, the sample needs to be taken as soon as possible and in any event within 24 h after death. The time since death may affect the reliability of the tests, hence the importance of using an ante-mortem sample whenever possible, unless the test has been validated for post-mortem blood samples.

In the case of neonate or infant donors less than 1 year of age, positive serological results (IgG) do not necessarily represent actual infection of the donor, as these antibodies can be passively transmitted and be of maternal origin. In such case, lack of the presence of IgM antibodies, and possibly additional testing using PCR, may allow to rule out the infection of the donor.

As stated in the regulations, if a transfusion was administered shortly before donation, haemodilution may have occurred, which needs to be taken into account because major dilution is liable to lessen the detectability of the antibodies or antigens in the donor blood. The results can also be invalid due to treatment with immunosuppressive agent (2006/17/EC).

Testing must be carried out on individual samples.

### Choice of additional biological tests

Apart from the mandatory tests, further tests may be decided upon as well. The decision whether or not to run optional tests depends on the type of tissue (infectious organisms may be present in some tissues and not in others) and the donor.

When tests are carried out on post-mortem samples the serological method used must have been validated for this type of samples by the producer or by the laboratory that performs the test in the event of there being no certification available from the producer.

Samples from deceased HBM donors need to be analysed using tests that have been validated by the producer or by the laboratory performing them in the event of there being no certification available from the producer for use on post-mortem samples.

### Test interpretation and implications for the releasing or rejecting of HBM

If the test results are positive for mandatory tests, the HBM will usually be rejected.

In some cases (e.g. a combination of positive results for anti-HBc and anti-HBs and negative results for HBsAg), the HBM can still be released, since this combination of results points to a past infection and immunity.

In some very specific cases, the results of a screening test may be overridden by a confirmatory test. This is only possible under certain conditions, which will be discussed below for each of the tests concerned.

In the case of autologous donations or donations between partners for medically assisted procreation (MAP) purposes, HBM from donors with positive test results may be released.

If viral inactivation steps are applied during processing (for example ensuring the safety of bone tissue), the serological results should be negative. So, as regard for living donors, NAT testing will not be performed and there is no need to repeat the test when the processing includes an inactivation step that has been validated for the infectious agents concerned.

As regards deceased donors, the tests to run include, at the very least: NAT screening for HIV, NAT screening for HCV and NAT screening for HBV, unless the processing includes an inactivation step that has been validated for the infectious agents concerned.

### Communicating the results of the tests

The royal decree of 28th of September 2009 setting the quality and safety norms indicates that the confirmed results of the donor assessment shall be communicated and clearly explained to the latter, taking into account the privacy requirements (Section 9, § 4, of the Act of 22 August** 2002). The information shall preferably be transferred via the attending physician or the general practitioner. The laboratory contact information shall be sent to the attending physician or general practitioner for any further questions regarding the results.

## Mandatory biological tests

The R.D. of 28 September 2009 mentioned above implementing a.o. the DIRECTIVE 2006/17/EC sets out mandatory biological tests that need to be performed on samples from HBM donors. At the very least, the following tests need to be performed:

- As regards living donors: anti-HIV 1,2; HBsAg, anti-HBc; anti-HCV and the syphilis screening test. When HBM from living donors that is intended for allogeneic use can be stored for extensive periods of time, repeat samples should be taken and retested after a period of 180 days. The repeat samples are aimed at reducing the window-phase of possible infection. If, in the case of a living donor, the sample of the donation, as defined above, it also tested by means NAT testing for HIV, HBV and HCV there is no need for repeat samples. Similarly, there is no need to repeat the test when the processing includes an virus inactivation step that has been validated for the infectious agents concerned.
- As regards deceased donors, the tests to run include, at the very least, those listed under paragraph A, as well as: NAT screening for HIV, HCV and for HBV, unless the processing includes an inactivation step that has been validated for the infectious agents concerned. Furthermore, back-screening of receptors of organs obtained from the same donor can provide additional safety.

Under certain circumstances, the donor history and specific features of the HBM intended for donation (e.g. malaria, CMV, toxoplasmosis) may require further testing. In part 3 of these recommendations, some of these optional tests including anti-CMV, anti-Toxoplasma, anti-EBV and anti-HTLV will be described more in detail.

### Biological tests for the detection of HIV antibodies/viral genome

#### HIV 1/2 antibodies (anti-HIV 1/2)

Serological screening tests for anti-HIV 1/2 are among the routine tests that are carried out in most diagnostic laboratories in Belgium. The strict regulations in place for the producers of these tests contributed to the placing on the Belgian market of high- performance and high-quality tests. As a result, there are usually no major difficulties when running or interpreting these tests.

Non-negative (grey area and reactive) screening results for anti-HIV-1/2) need to be confirmed by one of the recognised Belgian AIDS Reference Laboratories (ARLs).

Depending on the type of HBM donations, this most often primarily involves interpreting and reporting the results of the screening tests, given the fact that the confirmatory tests that are currently used cannot be carried out in an emergency situation.

**Fig. 1:**
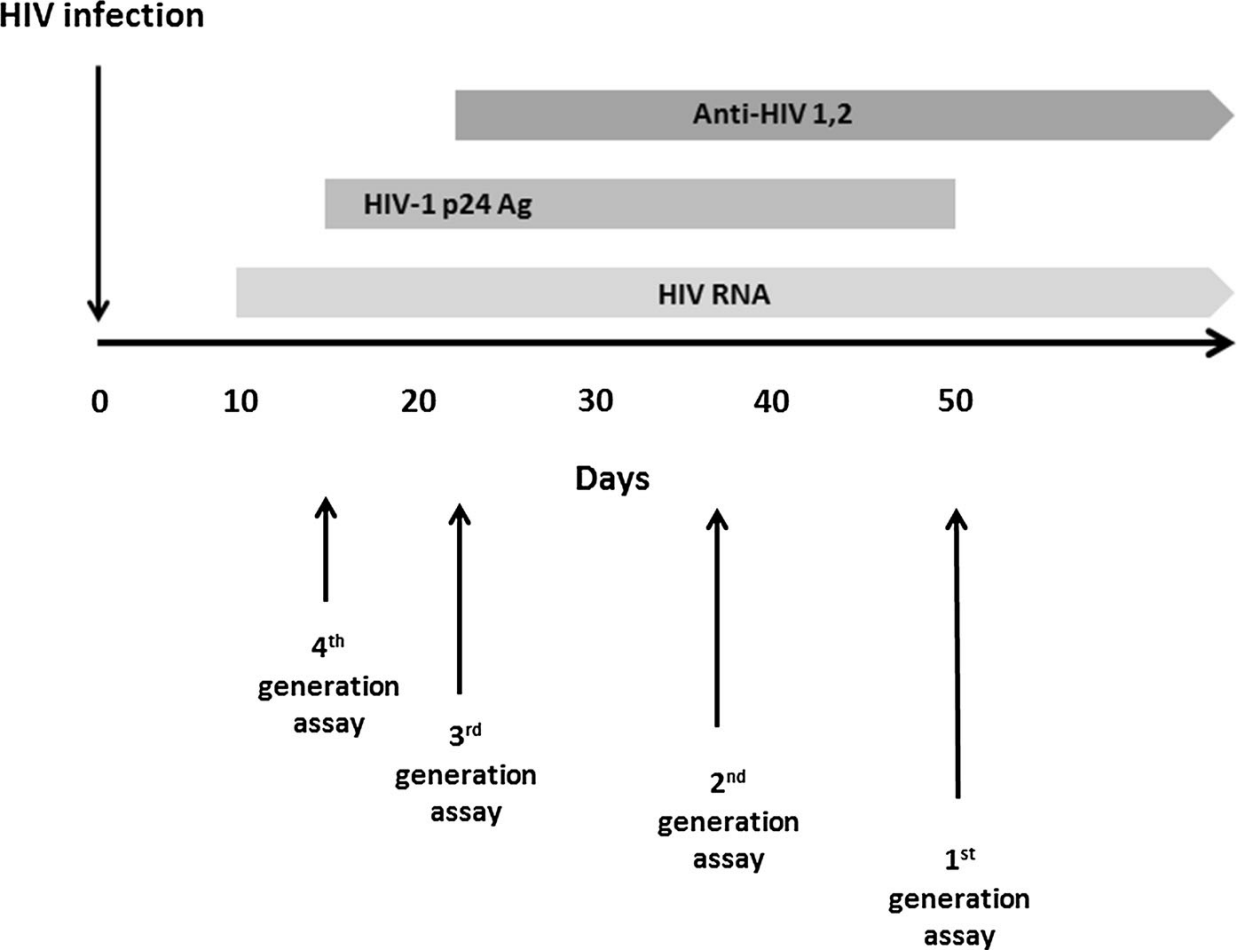
Serological profile in relation to “window”-periods for different generations of anti-HIV 1,2 assays. *Anti-HIV 1,2* antibodies against human immunodeficiency virus types 1, 2; *Ag* antigen

HIV 1/2-screening assays are traditionally subdivided into distinct “generations” according to the different principles on which they are based. As regards the serological immunoassays that are used for anti-HIV-1 screening, the general rule is that the higher the generation of the test (e.g. 4th generation), the shorter the serological “window period” between the infection with HIV and its detection (Fig. 1) (CDC 2014).

In Belgium most clinical diagnostic laboratories use 4th generation immunoassays. According to recent data on the External Quality Assessment (EQA) conducted by the Quality Department of the Medical Laboratories of the Scientific Institute of Public Health of 2016, 7/155 (4.5%) of Belgian diagnostic laboratories that took part in the EQA-cycle still use 3rd generation immunoassays. Immunoassays belonging to these two generations, 3rd and 4th, do not differ in terms of the detection of anti-HIV 1/2. However, 4th generation tests detect additionally p24 antigen of HIV which means that the HIV-1 infection can be detected before HIV antibodies can be detected.

It follows that using 3rd generation immunoassays for serological screening in HBM donors means that the “window period” will be 3–5 days longer compared to the use of 4th generation anti-HIV-1/2 tests.

Given the fact that the generation of the anti-HIV1/2 immunoassay used affects the “window period” of this test, which in turn impacts on the safety of the donation, it is of paramount importance to use the tests with the shortest “window period”, i.e. 4th generation tests.

In the event of non-negative (grey zone or reactive) screening result for anti-HIV 1/2, and pending confirmation by an ARL, the report must clearly state that these screening results still need to be confirmed. In Belgium, the ARL’s are in charge of interpreting and reporting the HIV confirmatory test results. The final result of the test is that obtained by an ARL on the basis of the confirmatory tests carried out.

#### HIV NAT testing

As was the case for serological assays, the analytical features of NAT tests used for HIV screening have evolved significantly over the past decade (Gullett and Nolte 2015; Hopkins et al. 2015). At the methodological level, almost every analytical feature of the NAT tests for HIV has been optimized. As with regard of the scope of the current recommendations, especially improvement in sensitivity and accuracy, also in low analytical range, is of importance.

Carrying out additional HIV NAT testing reduces the “window period” after a possible HIV infection with additional 3–4 days compared to the 4th generation anti-HIV 1/2 serological tests described above, which enhances the safety of HBM donation.

For the interpretation of the results of the biological tests for the detection of antibodies/RNA of HIV, see Table 1.

**Table 1:** Interpretation of the results of the biological tests for the detection of HIV antibodies/RNA

Anti-HIV 1/2^a^	HIV NAT	Interpretation	Consequence for the donation	Further management
Positive	Positive	Infection with a detectable viral load	Reject	Inform AP^b^
Positive	Negative	Infection with an undetectable viral load	Reject	Inform AP
Negative	Positive	Acute infection	Reject	Inform AP
Negative	Negative	No infection	Release possible	No further action

^a^Negative result in clinical diagnostic laboratory or confirmed negative/positive result in ARL

^b^*AP* attending physician

### Biological tests for the detection of HBV antibodies/DNA

#### HBV serology

The diagnosis of HBV infection is based upon interpretation of different serological markers. The fact that multiple markers are available and that these different markers display divergent patterns of appearance/persistence/disappearance during the period following the initial infection results in a fairly complex interpretation chart.

The R.D. of 28 September 2009 mentioned above provides that the serology for hepatitis B must, at the very least, include HBsAg and anti-HBc assays.

In addition, Annex VI, Section 1.3 of this R.D. states that if, on the one hand, the anti-HBc test is positive whilst on the other, the HBsAg and HBV NAT tests are both negative, an anti-HBs shall be carried out. If the latter test is positive, this points to a natural infection that has been cleared, which means that the positive anti-HBc test is not a contraindication for releasing the HBM for human application. This is a possible explanation for positive screening results for anti-HBc in areas with a low seroprevalence of HBsAg, as is the case in Belgium. Only tests measuring total anti-HBc are discussed in the current recommendations as the ones used in Belgian diagnostic laboratories.

If the HBsAg test is negative and the anti-HBc and anti-HBs tests are both positive, this may be due to a past naturally acquired HBV infection with HBsAg clearance.

However from a microbiological point of view, false positive result for anti-HBc cannot be excluded.

The serological profile corresponding to negative HBsAg and anti-HBs in combination with positive anti-HBc (“core only” HBV serology) is not explicitly described in the Belgian legislation. In this case the possible interpretations include naturally acquired HBV infection with clearance of HBsAg or false positivity for anti-HBc. Unlike the serological profile described above, there is no serological evidence in support of there being any immunity, as the anti-HBs test is negative. Despite the extremely low infectious risk associated with individuals with a “core only” serological profile (and negative HBV NAT), as a rule, HBM donations from these donors should nonetheless be rejected (CDC 2015a). In exceptional individual cases, the HBM administrator may derogate from this rule, but only after having consulted the laboratory virologist and after having carried out a thorough risk assessment in agreement with all the parties (including transplanting medical doctor and patient) concerned.

Carrying out additional HBV NAT testing has enhanced the safety of HBM donations, as these can yield positive results just 1 week after the initial infection, whilst screening based on the early serological marker for acute infection, viz. HBsAg, displays a high degree of variability and depends, among other things, on the viral inoculum, the route of transmission and host factors and usually only yields positive results at least 1 month after the initial infection. For donated HBM, it is usually possible to wait for the results of HBV NAT testing, and it is essential to interpret the serological results in conjunction with those of HBV NAT testing (Fornés et al. 2015; Workowski et al. 2015).

For the interpretation of the results of the biological tests for the detection of antibodies/DNA of HBV, see Table 2.

**Table 2:** Interpretation of the results of the biological tests for the detection of HBV antibodies/DNA

HBsAg	Total anti-HBc	Anti-HBs	HBV NAT	Interpretation	Consequence for the donation	Furthr management
Positive	Negative	Negative	Positive	Early acute infection	Reject	Inform AP^a^
Positive	Negative	Negative	Negative	Recent vaccination, acute infection or false-positive HBsAg result	Reject	Inform AP
Positive	Positive	Negative	Negative	Chronic^b^ infection with undetectable viral load	Reject	Inform AP
Positive	Positive	Negative	Positive	Acute/chronic^b^ infection with detectable viral load	Reject	Inform AP
Negative	Positive	Positive^c^	Negative	Past natural infection, immunity	Release possible	No further action
Negative	Positive	Negative	Positive	Occult^d^ infection	Reject	Inform AP
Negative	Positive	Negative	Negative	“core only” profile^e^	Reject	No further action
Negative	Negative	Positive	Negative	Post-vaccination immunity	Release possible	No further action
Negative	Negative	Negative	Positive	Acute infection or false-positive result for HBV NAT	Reject	Inform AP

^a^*AP* attending physician

^b^Chronic infection is defined as persistence of HBsAg for > 6 months

^c^≥ 10 International Units (IU)/liter (l) (Morbidity and Mortality Weekly Report 2011; Derouin et al. 2008)

^d^Occult infection is defined as negative result for HBsAg with presence of HBV DNA in blood or tissues

^e^See further in the text interpretation of “core only” serological profile

### Biological tests for the detection of HCV antibodies/RNA

#### Anti-HCV

The most recent guidelines from both the Centers for Disease Control and Prevention (CDC 2015b) and the European Association for the Study of Liver (EASL 2018), favor anti-HCV as the first-line diagnosis of an HCV infection. In the event of positive result for anti-HCV, it is advised by both international guidelines to search for HCV RNA.

Interpretation difficulties are mainly due to the combination of a positive result for anti-HCV and a negative result for HCV RNA.: this serological profile may be attributable to a past HCV infection that has subsequently cleared or to a false-positive anti-HCV result.

Ideally, one of the following confirmatory strategies will be chosen in the event of a suspected false-positive result for HCV antibodies. The second strategy is hereby the preferable one to confirm that this is indeed a false-positive result.

1. the carrying-out of an additional serological assay for anti-HCV based on a different method than the original test. Repeat false-positive results are known to be rather unlikely, especially if different methods are used. Also, if additional testing yields a negative result, this indicates that the result for the initial serological assay was false-positive. Given the fact that these different serological methods also vary in terms of their specificity, the competent laboratory virologist needs to be consulted.
2. if the initial serological assay yields a low analytical value, the result will probably be false-positive. For the vast majority of serological tests for anti-HCV, the analytical results will be expressed as S/CO (Signal to cut-off ratios). For some anti-HCV tests, the CDC have suggested an analytical value below which the result can be regarded as probably false-positive. For the most recent anti-HCV tests, however, such a cut-off value for potentially false-positive results has not (yet) been verified.

#### HCV NAT testing

Given the fact that a considerable amount of time elapses between the initial infection and the appearance of anti-HCV, the use of HCV NAT testing has enhanced the safety of HBM donations. It is estimated that in about half of those with an acute HCV infection and detectable HCV RNA, the search for anti-HCV remains negative.

For the interpretation of the results of the biological tests for the detection of HCV antibodies/RNA, see Table 3.

**Table 3:** Interpretation of the results of the biological tests for the detection of HCV antibodies/RNA

Anti-HCV	HCV NAT	Interpretation	Consequence for the donation	Further management
Positive	Positive	Infection with a detectable viral load	Reject	Inform AP^a^
Positive	Negative	Passed infection with an undetectable viral load or false-positive result for anti-HCV	Reject^b^	Inform AP
Negative	Positive	Early infection	Reject	Inform AP
Negative	Negative	No infection	Release possible	No further action

^a^*AP* attending physician

^b^In the event of a low analytical value, the result will probably be false-positive. For the vast majority of serological tests for anti-HCV, the analytical results will be expressed as S/CO. For some anti-HCV tests, the CDC have suggested an analytical value below which the result can be regarded as probably false-positive. For the most recent new anti-HCV tests, however, such a cut-off value for potentially false-positive results has not (yet) been verified (CDC 2015b)

### Biological screening tests for syphilis

Recent epidemiological findings confirm that syphilis is more common in people who have also contracted HIV and hepatitis C. Conversely, increasingly sophisticated serological and NAT tests have become available in recent years to screen for HIV and hepatitis B and C. This has allowed to shorten the window period to such an extent that a positive syphilis test retains but a very limited relative value as an indicator for an increased risk of HIV and hepatitis C (Marrazzo 2014; Gallego-Lezaun et al. 2015; Jansen et al. 2015; Peterman et al. 2015; Reisner et al. 2015).

A validated testing algorithm must be applied to exclude the presence of active infection with Treponema pallidum. A non-reactive test, specific or non-specific, can allow tissues and cells to be released. When a non-specific test is performed, a reactive result will not prevent procurement or release if a specific Treponema confirmatory test is non-reactive. A donor whose specimen tests reactive on a Treponema-specific test will require a thorough risk assessment to determine eligibility for clinical use (EU 2006/17).

#### Serological tests for the detection of syphilis

The algorithms that are recommended for the serological investigation for syphilis are challenging due to the inherent complexity of the applied methods. In the serological diagnostics of syphilis classification in non-treponemal and treponemal tests is used with frequently challenging interpretations and need for the supplementary confirmatory testing (Morshed 2014).

Non-treponemal tests are tests that search for IgG and IgM directed against the lipids that are released from the damaged human cells during an early stage of the disease. Their goal is therefore to search for antibodies to antigens that are not specific to an infection with species of the genus *Treponema*, as reflected in the term reaginic antibodies. The non-specific nature of this category of serological tests is also reflected in the fact that many other causes, such as advanced age, pregnancy, various types of malignant tumors, autoimmune diseases and other unrelated infections may result in the formation of anti-lipoid antibodies, thus generating false-positive results. Consequently, a positive result obtained with a non-treponemal test should always be confirmed by means of a treponemal test. Moreover, non-treponemal tests usually display a low sensitivity as regards the detection of early syphilis, whilst the first positive results are only obtained some 4–8 weeks after infection. The tests belonging to this category have mainly a diagnostic purpose as part of the therapeutic follow-up of patients with syphilis. Thus, a declining titre over a certain period of time is indicative of a favourable response to treatment. As a rule, a successful treatment leads to negative results for these tests. The Venereal Diseases Research Laboratory (VDRL) test and Rapid Plasma Reagin (RPR) test belong to this group of non-treponemal tests used for serological syphilis screening.

Treponemal tests are serological screening tests that search for specific antibodies directed against species of the genus Treponema. No distinction can be made between the different treponematoses due to immunological cross-reactions. These tests usually remain positive after the initial infection, which means that they cannot be used to monitor the response to treatment or diagnose reinfections. Treponemal serological tests include the *T .pallidum* haemagglutination (TPHA) test, the *T. pallidum* particle agglutination (TPPA) test, treponemal enzyme immunoassays (EIA), chemiluminescence immunoassays (CLIA) and immunoblotting.

New developments, especially as regards the optimisation of treponemal immunoassays, offer new possibilities due to the earlier detection of syphilis and the shorter diagnostic window, but do not necessarily simplify the assessment of the overall serological picture.

According to recent international recommendations (IUSTI 2014), different screening algorithms can be used for serological syphilis screening:

- *Only the treponemal screening test* This screening strategy is commonly used in European blood banks and laboratories due to its potential for large-scale automation. This algorithm identifies both individuals in whom syphilis has been treated successfully as well as those who have not received any treatment. It is better suited to detect the early stages of infection than the sole use of a non-treponemal test. Given the fact that this strategy is mainly used for populations with a low prevalence of syphilis, it is fraught with a considerable number of false-positive results.
- *Only the non-treponemal screening test* Ideally, a non-treponemal test carried out for screening purposes should be quantitative in nature in order to rule out the prozone effect when using undiluted blood samples (this concerns < 2% of samples, usually during the secondary phase of syphilis. These patients display extremely high titres of antibodies that interfere with the formation of antigen–antibody complexes, which are necessary to visualise flocculation when interpreting the non-treponemal test). This algorithm can only detect active (infectious) syphilis, which means that it can miss the early stage of syphilis.
- *Treponemal and non-treponemal tests* This algorithm is especially useful to screen high-risk populations as well as to screen for the early stages of syphilis.

In the serological diagnosis of syphilis and independently of the screening algorithm used, a confirmatory test will always need to be carried out, regardless of which of the screening tests turned out positive.

- If the initial screening test only included a treponemal test, the results should be confirmed by means of a second treponemal test based on a different analytical method as well as a quantitative non-treponemal test if this second treponemal test also turns out positive.
- If the initial screening test only included a non-treponemal test, the positive result needs to be confirmed by means of a treponemal test, whereas the non-treponemal test should be performed in a quantitative manner if this was not initially the case.
- If the initial screening was performed using a treponemal test as well as a non-treponemal test, the non-treponemal test should be performed in a quantitative manner. A second treponemal test based on a different analytical method may be used to rule out a false-positive result for the initial treponemal test only if the non-treponemal test is negative.

It seems advisable to carry out treponemal tests (alone or in combination with non-treponemal tests) during the initial screening that is carried out as part of the process of HBM donations to ensure maximum safety for the HBM intended for donation.

For the interpretation of the results of the biological tests for the serological detection of syphilis, see Table 4.

**Table 4:** Interpretation of the results of the biological tests for the serological detection of syphilis

Treponemal test	Non-treponemal test	Interpretation	Consequence for the donation	Further management
Positive	Positive^a^	Active infection	Reject	Inform AP^b^
Positive	Negative	(Treated) past infection or early infection of false-positive treponemal test	Reject^d^	Inform AP^c^
Negative	Not carried out or negative	No infection	Release possible	No further action
Negative	Positive	False-positive result for the non-treponemal test or false-negative result for the treponemal test	Release potentially possible^d^	No further action

^a^Given the fact that in the vast majority of cases in which non-treponemal tests yielded false-positive results, the titre were ≤ 1/4; a “positive non-treponemal test” is considered to be a test with a titre of ≥ 1/8

^b^*AP* attending physician

^c^In this serological situation confirmatory treponemal test should be performed. In case of negativity of confirmatory treponemal test, is the initial positive screening for treponemal test seen as false positive, justifying HBM release; no contact with AP is required

^d^The bank administrator can still accept the HBM after having consulted the pathologist, possible after carrying out additional tests (quantitative non-treponemal test; additional treponemal test) and after having received the informed consent of the recipient and the medical transplant team

## Optional biological tests

Based on the European Directives a number of tests are mandatory however the application in different European countries is variable as demonstrated in *EU mapping of blood donor testing requirements 2015*. From this survey Belgium is a safe country for HBM transplantation. Optional tests can always be performed but the implication on the availability of HBM should be kept in mind.

The R.D. of 28 September 2009 mentioned above provides that further biological testing is required under specific circumstances. The criteria that are crucial when selecting possible additional tests have to do with the donor’s medical history and specific features of the donated HBM. This paper looks at screening for HTLV-1 antibodies, CMV, toxoplasmosis and EBV as examples for such additional serological tests.

The decision to run these optional tests is made on the basis of the tissues and cells that are intended for donation as well as specific clinical and epidemiological circumstances.

Infections are transmitted most effectively via viable cells and tissues, blood, stem cells and vascularized organs. In addition the risk might be greater if the recipient is immunocompromised and the HBM has not undergone a decontamination procedures (Fishman et al. 2012).

### Biological tests for the detection of antibodies/DNA of *Toxoplasma gondii*

#### Anti-toxoplasma

Serological tests for the detection of anti-Toxoplasma IgG and IgM belong to routine testing in the most clinical diagnostic laboratories in Belgium. There is a profound commercial offer available on the diagnostic market. In general, the anti-Toxoplasma IgG does not pose any significant analytical problems, whereas the search for anti-Toxoplasma IgM is subject to possible cross-reactivity with other acute unrelated infectious processes (e.g. acute CMV or EBV infections) or autoimmune diseases outcoming in false positive results (Roberts et al. 2001; Kodym et al. 2007). In recent years, laboratories have acquired considerable expertise in the use of *Toxoplasma* IgG-avidity tests that are not fraught with specificity problems that are typical of IgM assays (Lappalainen and Hedman 2004; Sensini 2006; Candolfi et al. 2007; Villard et al. 2013).

Molecular assays for the identification of *Toxoplasma* DNA are rarely, if at all, performed as part of the HBM donation process in particular and in general for the confirmation of active infection in Belgian clinical diagnostic laboratories. Since requests for *Toxoplasma gondii* genome screening are very rare, this assay is not widely available in Belgian diagnostic laboratories. It is almost exclusively carried out as part of the screening/confirmatory process of ocular or congenital toxoplasmosis.

For the interpretation of the results of the biological tests for the detection of antibodies against *Toxoplasma gondii*, see Table 5.

**Table 5:** Interpretation of the results of the biological tests for the detection of antibodies against *Toxoplasma gondii*

Anti-toxoplasma IgG	Anti-toxoplasma IgM	Interpretation	Consequence for the donation	Further management
Positive	Positive	Acute infection or false-positive IgM result	Reject	Inform AP^a^
Negative	Positive	Acute infection or false-positive IgM result	Reject	Inform AP
Positive	Negative	Past infection	Release possible	No further action
Negative	Negative	No infection	Release possible	No further action

^a^*AP* attending physician

### Biological tests for the detection of EBV antibodies/DNA

#### Anti-EBV

**Fig. 2:**
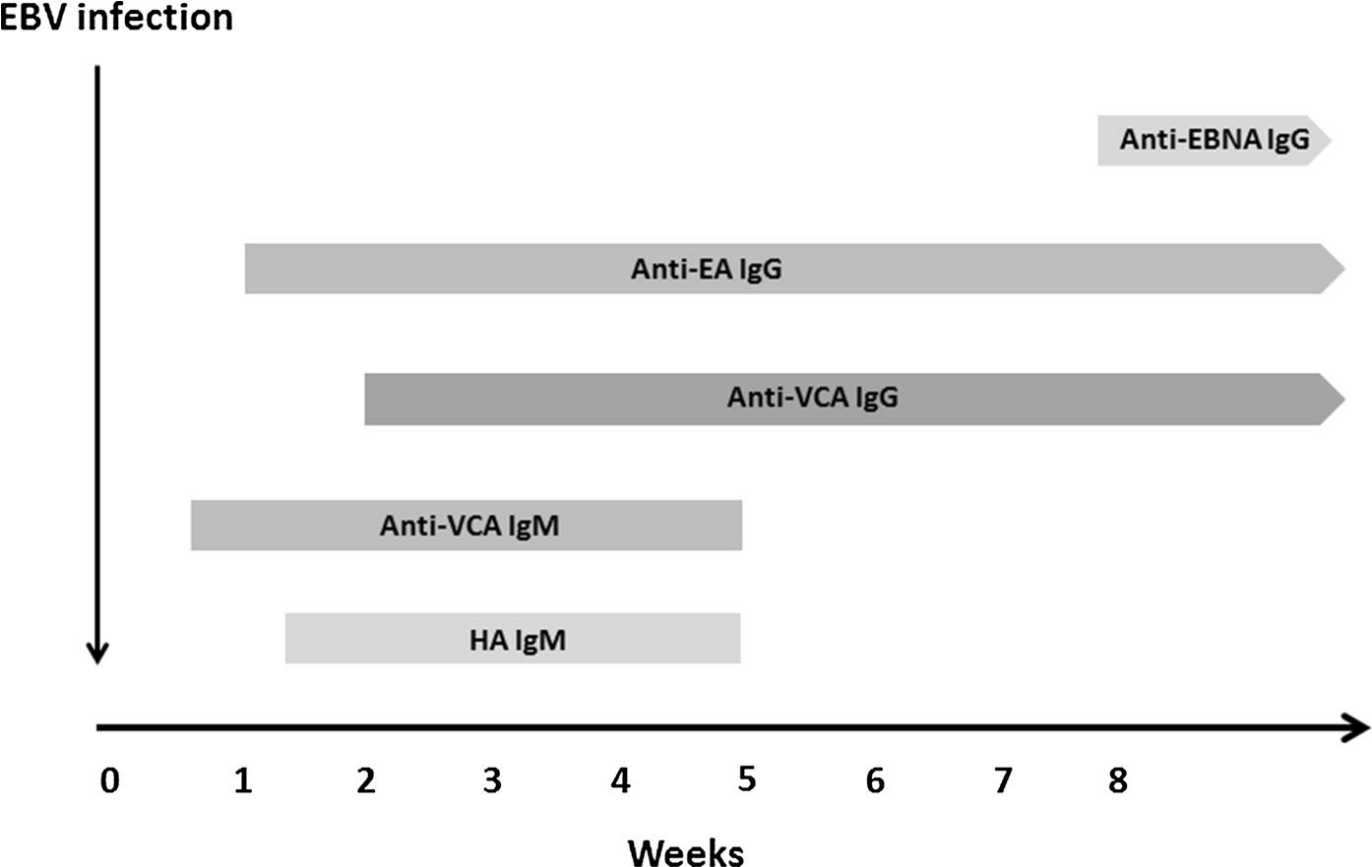
Serological progression of EBV infection in relation to detection of antibodies to different EBV antigens. *HA IgM* heterophilic IgM antibodies; *anti-VCA IgM* IgM antibodies against viral capsid antigen; *anti-VCA IgG* IgG antibodies against viral capsid antigen; anti-EBNA IgG IgG antibodies against EBV nuclear antigen; *anti-EA IgG* IgG antibodies against EBV early antigen

Most Belgian diagnostic laboratories already routinely use serological testing to screen for both specific and non-specific antibodies to EBV. There is a broad commercial offer available that allows to search for a wide range of parameters, thus contributing to a more accurate interpretation. These parameters as well as the testing algorithms differ from one diagnostic laboratory to another. The following serological parameters can be searched for in order to establish the serological progress of the EBV infection and especially to detect/rule out an acute infection: non-specific heterophile IgM antibodies (anti-HA IgM), IgM antibodies to viral capsid antigens (anti-VCA IgM), IgG antibodies to viral capsid antigens (anti-VCA IgG), IgG antibodies to EBV nuclear antigens (anti-EBNA IgG) and IgG antibodies to EBV early antigens (anti-EA IgG) (Fig. 2). The commercially available serological tests that are commonly used to search for anti-EBV IgG do not present any significant analytical problems, whereas anti-EBV IgM screening tests have to contend with a considerable number of false-positive results, mainly as regards patients with acute infections with taxonomically related herpes viruses (e.g. CMV, Varicella Zoster virus). (Gulley and Tang 2008; Neocleous et al. 2013; De Paschale and Clerici 2012).

Molecular assays for the identification of EBV DNA are rarely, if ever, performed as part of the HBM donation process. The same is true for Belgian diagnostic laboratories. These assays are usually only carried out as part of the screening and follow-up process of patients who are at an increased risk of post-transplant lymphoproliferative disease (PTLD).

Each individual diagnostic laboratory is responsible for determining which test combinations they will use to detect acute/past EBV infections and reactivations. The tests mentioned above do not always need to be carried out.

For the interpretation of the results of the biological tests the detection of antibodies against EBV, see Table 6.

**Table 6:** Interpretation of the results of the biological tests for the detection of antibodies against EBV

Heterophile antibodies	Anti-VCA IgM	Anti-VCA IgG	Anti-EBNA IgG	Anti-EA IgG	Interpretation	Consequence for the donation	Further management
Positive	Positive	Negative	Negative	Positive	Early acute infection	Reject^a^	Inform AP^b^
Positive	Positive	Positive	Negative	Positive	Acute infection/early recovery	Reject^a^	Inform AP
Positive	Negative	Negative	Negative	Negative	Early acute infection or false-positive result for heterophile antibodies	Reject^a^	Inform AP
Negative	Positive	Negative	Negative	Negative	Early acute infection or false-positive result for anti-VCA IgM	Reject^a^	Inform AP
Negative	Negative	Positive	Negative	Positive	Recovery	Release possible	No further action
Negative	Negative	Positive	Positive	Positive	Recovery	Release possible	No further action
Negative	Negative	Positive	Positive	Negative	(past) infection	Release possible	No further action

^a^Only if seronegative recipient

^b^*AP* attending physician

### Biological tests for the detection of HTLV

Directive 2012/39/EU requires that HTLV-1 antibody tests be carried out in certain circumstances: in the case of living donors living in or originating from regions with a high prevalence or whose sexual partner or parents come from such regions.

Most of the serological tests that are currently available on the market detect anti-HTLV-1 and HTLV-2 antibodies. They use recombinant antigens and/or synthetic peptides, which enhances their specificity compared to that of first generation tests. Nevertheless, the positive predictive value of these tests remains low, especially in low-prevalence populations. A confirmatory immunoblot must therefore be carried out for all positive screening tests. A positive immunoblot confirms the diagnosis, whilst a negative immunoblot rules out an HTLV-1/2 infection. In some cases, the result may be indeterminate, i.e. there is reactivity to one or several HTLV-antigens, whilst the criteria for positivity are not met.

This is a common situation (up to 50% of cases) in endemic areas and may indicate early seroconversion. It rarely occurs in Belgium, which has a very low seroprevalence (< 0.1%) (Costa et al. 2011; ECDC 2012a, b; Marano et al. 2016).

In this respect, molecular biology techniques (NAT tests) play only a very marginal role, as the latter do not confer any advantages in terms of sensitivity, specificity and cost.

Some studies reveal that the sensitivity of the immunoblot is superior to that of NAT testing in the diagnosis of HTLV-1/2 infection in the event of a positive screening test result. One explanation for the lower sensitivity of NAT testing in this context is the low viral load displayed by some asymptomatic individuals (Costa et al. 2011).

For the interpretation of the results of the biological tests for the detection of anti-HTLV-1, see Table 7.

**Table 7:** Interpretation of the results of the biological tests for the detection of antibodies against HTLV-1

Anti-HTLV-1 screening	Anti-HTLV-1 immunoblot	Interpretation	Consequence for the donation	Further management
Negative	Not carried out	No infection	Release possible	No further action
Positive	Negative	No infection	Release possible	No further action
Positive	Indeterminate	Possible infection	Reject	Inform AP^a^
Positive	Positive	Infection	Reject	Inform AP

^a^*AP* attending physician

### Biological tests for the detection of CMV

During CMV primary infection, the virus spreads via polymorphonuclear cells and monocytes and afterwards remains for life, mainly in endothelial cells, bone-marrow progenitor cells, and circulating monocytes. Under certain circumstances, such as the stem cell or sperm donations, serological testing for CMV is required. The tests used detect anti-CMV IgG and IgM.

Screening for anti-CMV IgG allows to identify donors carrying CMV, which is true for 50% of the population in countries with high socio-economic standards such as Belgium. Detecting anti-CMV IgG poses few interpretation problems, except for equivocal results, i.e. in the grey zone of the test. In that case, it is possible to run a second IgG test, viz. one that is based on a different analytical method, but if any doubt remains, the donor should be considered as CMV positive.

IgM tests are less specific and their interpretation often poses problems when there is no suggestive clinical presentation. Indeed, the presence of anti-CMV IgM is not necessarily linked to a recent infection. Positive results can also be due to cross-reactions with other herpes viruses such as Epstein–Barr virus or another infection leading to non-specific polyclonal stimulation of the immune system. In addition, the use of increasingly sensitive techniques makes it possible to detect specific IgM antibodies long after the onset of primary infection (FDA 2007; Kotton et al. 2013).

Analogous with use of Toxoplasma IgG avidity testing, avidity testing for CMV IgG can be applied in certain clinical situations in case of positivity for anti-CMV IgG as well as anti-CMV IgM. Current poor standardization of avidity testing for CMV IgG has to be kept in mind (Revello et al. 2010).

In case of doubt, confirmation using molecular techniques seems justified, given the fact that clinical laboratories are experienced in carrying out molecular assays on blood/plasma samples, though not as part of the diagnosis of acute infection. These findings imply that it is necessary to consult with the competent laboratory virologist if the decision is made to carry out a molecular assay.

For the interpretation of the results of the biological tests the detection of antibodies against CMV, see Table 8.

**Table 8:** Interpretation of the results of the biological tests for the detection of antibodies against CMV

Anti-CMV IgG	Anti-CMV IgM	Interpretation	Consequence for the donation	Further management
Positive	Positive	Acute infection or false-positive result for IgM	Release possible^a^	Inform AP^b^
Negative	Positive	Acute infection or false positive result for IgM	Release possible^a^	Inform AP
Positive	Negative	Old infection	Release possible	No futher action
Negative	Negative	No infection	Release possible	No further action

^a^Depending on the type of donation and the serostatus of the recipient

^b^*AP* attending physician

### Other tests

Depending on the travel history and specific current or past clinical situation of the HBM donor as well as the ongoing epidemiological situation and the nature of the HBM intended for donation, the decision can be made to carry out other optional tests, such as screening for tropical infections such as malaria, trypanosomiasis, infections with the West Nile virus, Zika virus, etc. The need to perform such assays, or even others, must be examined on a case-by-case basis.

## Discussion

A number of practical recommendations have been formulated with the intention to make progress in the standardization of in particular testing, interpretation of biological tests on blood samples of potential donors of human body material. Better and more standardized interpretation of sometimes challenging results of biological tests will facilitate their use, promote communication between banks of human body material and clinical diagnostic laboratories, and will finally increase the safety of donation. On the other hand the correct evaluation of (a combination of) results of biological tests will diminish the number of rejected potential donors due to too strict interpretation of individual tests.
