# Correction to: Biological tests carried out on serum/plasma samples from donors of human body material for transplantation: Belgian experience and practical recommendations

**DOI:** 10.1007/s10561-018-9728-8

**Published:** 2018-10-26

**Authors:** Elizaveta Padalko, Katrien Lagrou, Marie-Luce Delforge, Hilde Jansens, Nadine Ectors, Jean-Paul Pirnay, Johan Klykens, Etienne Sokal, Ludo Muylle, Agnes Libois, Alain Vanderkelen, Gilbert Verbeken, Conny Matthys, Dominique Goossens, Geert Hanssens, Muriel Baltes, Hilde Beele

**Affiliations:** 1 Department of Clinical Chemistry, Microbiology and Immunology, Ghent University/University Hospital, De Pintelaan 185, 2P8, 9000 Ghent, Belgium; 2 School of Life Sciences, Hasselt University, Agoralaan Building D, 3590 Diepenbeek, Belgium; 3 Working Group on Cells, Tissues and Organs of the Superior Health Council of Belgium, Brussels, Belgium; 4 KU Leuven and University Hospitals of Leuven, Herestraat 49, 3000 Louvain, Belgium; 5 Université Libre de Bruxelles/Hopital Erasme, Route de Lennik 808, 1070 Brussels, Belgium; 6 Antwerp University and Antwerp University Hospital, Wilrijkstraat 10, 2650 Edegem, Belgium; 7 Laboratory for Molecular and Cellular Technology, Queen Astrid Military Hospital, Bruynstraat 1, 1120 Brussels, Belgium; 8 Centre de Thérapie Cellulaire, Cliniques Universitaires St Luc, Université Catholique de Louvain, 10 av Hippocrate, B 1200 Brussels, Belgium; 9 CHU Saint-Pierre, Université Libre de Bruxelles, 322 rue haute, 1000 Brussels, Belgium; 10 Red Cross, Namur, Belgium; 11 Sint-Genesius-Rode, Belgium

## Correction to: Cell Tissue Bank 10.1007/s10561-018-9721-2

The article Biological tests carried out on serum/plasma samples from donors of human body material for transplantation: Belgian experience and practical recommendations, written by Elizaveta Padalko, Katrien Lagrou, Marie-Luce Delforge, Hilde Jansens, Nadine Ectors, Jean-Paul Pirnay, Johan Klykens, Etienne Sokal, Ludo Muylle, Agnes Libois, Alain Vanderkelen, Gilbert Verbeken, Conny Matthys, Dominique Goossens, Geert Hanssens, Muriel Baltes and Hilde Beele, was originally published electronically on the publisher’s internet portal (currently SpringerLink) on 29 August 2018 without open access.

With the author(s)’ decision to opt for Open Choice the copyright of the article changed on October 2018 to © The Author(s) 2018, and the article is forthwith distributed under the terms of the Creative Commons Attribution 4.0 International License (http://creativecommons.org/licenses/by/4.0/), which permits use, duplication, adaptation, distribution and reproduction in any medium or format, as long as you give appropriate credit to the original author(s) and the source, provide a link to the Creative Commons license and indicate if changes were made.

